# A181 INFECTION WITH THE RAT TAPEWORM *HYMENOLEPIS DIMINUTA* REVEALS AN INTERLEUKIN-4 INDEPENDENT TUFT CELL ASSOCIATED WITH PEYER’S PATCHES

**DOI:** 10.1093/jcag/gwab049.180

**Published:** 2022-02-21

**Authors:** S Li, S Rajeev, A Wang, D M Mckay

**Affiliations:** department of Physiology and Pharmacology, University of Calgary, Calgary, AB, Canada

## Abstract

**Background:**

Infection with the rat tapeworm *Hymenolepis diminuta* elicits a T helper 2 (Th2) imunity and suppresses dinitrobenzene sulphonic-acid-induced colitis in mice. However, the signaling cascade in an immunocompetent host that recognizes and mobilizies early immune events to expel the worm is poorly understood. To fully understand how helminth-infection can ameliorate concomitant disease, it is important to elucidate key cells/mediators in the detection of the worm and early events in the local anti-worm response. Here, we assess the chemosensory epithelial tuft cell and Peyer’s patches (PP) as the primary inductive sites of mucosal immunity in *H. diminuta*-infected mice.

**Aims:**

To assess the role of tuft cells in PP development and worm expulsion following infection of *H. diminuta*.

**Methods:**

BALB/c, BALB/c *Il-4receptor-a*^*-/*-^, C57Bl/6 and C57Bl/6 *pou2f3*^*-/*-^ (transcription factor critial for tuft cell development) mice were infected with 5 cysticercoids of *H. diminuta* and necropsied 5-, 8- and 11-days post-infection (dpi); time-matched non-infected mice served as controls. PP number and size were counted and measured. Enteric tuft cells were assessed by immunostaining for the canonical marker double cortin like kinase (dclk)-1, and enumerated in PP-associated epithelium and villous epithelium distant from PP.

**Results:**

Dclkl^+^ tuft cells were sparse throughout villus epithelium of control mice, but were notably aggregrated over PPs. Infection with *H. diminuta* resulted in more detectable PPs by visual inspection, but did not result in a statistically significant increase in either size or number of PP. Infected mice showed increased numbers of dclk1^+^ tuft cells in villus epithelium and PP-associated epithelium (n=3–6). Tuft cells were absent in *pou2f3*^*-/*-^ mice, that showed normal size and number of PPs ± infection with *H. diminuta*. Unexpectedly, analysis of small intestine from *il4ra*^*-/*-^ mice revealed dclk1^+^ tuft cells in association with PPs; perhaps a unique sub-type of this sentinel cell.

**Conclusions:**

Analysis of tuft cells after infection with *H. diminuta* revealed that these cells aggregate around PPs under homeostatic conditions, and future studies will explore if this distrirbution is important for ‘M’ cell development and/or PP immune reactivity. Intrigingly, an *il-4ra*-independent tuft cell subtype was observed that awaits fuller characterization. Understanding the tuft cell may reveal novel aspects of development of mucosal immunity relevant to combating helminth-infection and perhaps autoinflammatory enteric disease.

**Funding Agencies:**

NSERC

